# Cell Homing Strategies in Regenerative Endodontic Therapy

**DOI:** 10.3390/cells14030201

**Published:** 2025-01-29

**Authors:** David Kim, Sahng G. Kim

**Affiliations:** 1 Center for Dental and Craniofacial Research, Columbia University College of Dental Medicine, New York, NY 10032, USA; dakim3124@gmail.com; 2 Division of Endodontics, Columbia University College of Dental Medicine, New York, NY 10032, USA

**Keywords:** regenerative endodontics, pulp regeneration, pulp revascularization, pulp revitalization, cell homing, cell free, signaling molecules, biomaterial scaffolds

## Abstract

Cell homing, a process that leverages the body’s natural ability to recruit cells and repair damaged tissues, presents a promising alternative to cell transplantation methods. Central to this approach is the recruitment of endogenous stem/progenitor cells—such as those from the apical papilla, bone marrow, and periapical tissues—facilitated by chemotactic biological cues. Moreover, biomaterial scaffolds embedded with signaling molecules create supportive environments, promoting cell migration, adhesion, and differentiation for the regeneration of the pulp–dentin complex. By analyzing in vivo animal studies using cell homing strategies, this review explores how biomolecules and scaffold materials enhance the recruitment of endogenous stem cells to the site of damaged dental pulp tissue, thereby promoting repair and regeneration. It also examines the key principles, recent advancements, and current limitations linked to cell homing-based regenerative endodontic therapy, highlighting the interplay of biomaterials, signaling molecules, and their broader clinical implications.

## 1. Introduction

Dental pulp tissue plays a crucial role in maintaining the vitality and function of teeth [1,2]. It consists of connective tissue, blood vessels, nerves, and a variety of cells, including fibroblasts, odontoblasts, and immune cells [1,2,3,4]. This highly specialized tissue provides nutrients, immune defense, and sensory function to the tooth [2]. However, dental pulp is susceptible to injury from trauma and infection. When it is damaged, its ability to self-repair is limited, often requiring therapeutic intervention to avoid further complications, including tooth loss.

Traditional approaches to managing pulp injuries, such as root canal therapy, are effective in resolving pain and eliminating infection [5]. However, these methods do not restore the biological and functional properties of the pulp–dentin complex. As a result, the tooth becomes devitalized, more prone to further damage, and reliant on restorative materials [5]. The advent of regenerative endodontics has introduced innovative techniques aimed at restoring the natural structure and function of the dental pulp [6]. Among these, cell homing-based strategies have emerged as a minimally invasive and biologically driven alternative [6].

Cell homing refers to the recruitment of the body’s own stem/progenitor cells to the site of injury [7]. This process relies on chemotactic gradients established by signaling molecules, such as growth factors and cytokines, which attract cells capable of regenerating the damaged tissue. This approach contrasts with cell transplantation methods, which involve isolating, expanding, and transplanting stem cells in the affected area. While cell transplantation has shown promise in preclinical and clinical studies, it faces challenges, including immunorejection, high costs, and regulatory hurdles [7]. In contrast, cell homing utilizes the body’s endogenous healing mechanism, offering a more translatable and scalable solution [7].

The development of biomaterials has further advanced the field of cell homing-based regenerative approaches. Scaffolds and matrices designed to mimic the natural extracellular matrix (ECM) of dental pulp provide structural support and biochemical cues essential for cell migration, adhesion, and differentiation [8,9]. Innovations in material science, such as synthetic polymers and self-assembly peptides, have led to the creation of biomaterials capable of delivering signaling molecules in a controlled manner [10,11]. These materials not only enhance the recruitment of endogenous cells but also create a conducive environment for tissue regeneration.

Signaling molecules are integral to the cell homing process. These factors establish chemotactic gradients that guide stem cells to the site of infection or injury [12,13]. In addition, they promote angiogenesis, neurogenesis, and cell differentiation, all of which are essential for the functional restoration of dental pulp [14]. The combination of biomaterials and signaling molecules has demonstrated significant potential in preclinical studies, showing improved outcomes in terms of vascularization, mineralization, and the formation of pulp–dentin-like tissues.

Several reviews have discussed the cell homing approach for regenerative endodontic therapy [15,16,17,18]. This review incorporated more recent studies, adhering to specific eligibility criteria compared to previous reviews. By analyzing a larger dataset, this review offers more current insights into tissue engineering protocols aimed at the clinical translation and applicability of a cell homing-based approach for dental pulp regeneration. This review aims to delve into the fundamental principles, recent advancements, and challenges associated with cell homing-based regenerative endodontic therapy. The integration of biomaterials, signaling molecules, and clinical implications is critically analyzed, providing a comprehensive overview for researchers and clinicians in the field of regenerative dentistry.

## 2. Materials and Methods

A comprehensive electronic search was conducted across PubMed, Scopus, Web of Science, Embase, and Medline databases from their inception to November 2024. The search utilized specific keyword combinations, including “pulp regeneration”, “pulp revascularization”, “pulp revitalization”, “regenerative endodontics”, “animal”, “pulpectomy”, “cell free”, and “cell homing.” In addition, manual searches were performed by reviewing the reference lists of the selected articles. The articles were screened based on the following inclusion or exclusion criteria. Inclusion criteria encompassed animal studies, pulpectomy performed, the application of signaling molecules or biomaterials containing signaling molecules within the root canal space, and orthotopic models. Exclusion criteria ruled out in vitro studies, clinical studies, review articles, studies involving cells transplanted into the root canal space, ectopic models, and studies that did not perform histology. Studies were identified by an initial screening based on titles and abstracts followed by a thorough evaluation of the full texts to determine their eligibility for inclusion in the review. Other pertinent studies investigating the biological effects of signaling molecules and biomaterial scaffolds and the mechanism of cell homing within the context of regenerative endodontic therapy were also incorporated to broaden and enrich the scope of this narrative review.

## 3. Mechanism of Cell Homing

**Figure 1:**
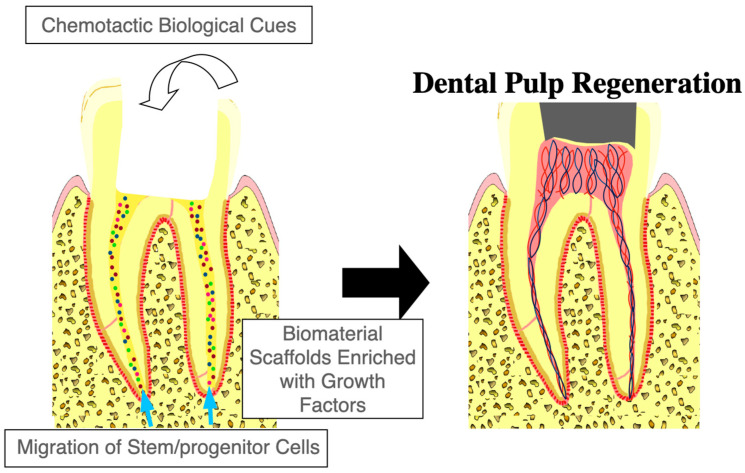
Cell homing-based regenerative endodontic therapy. Cell homing-based regenerative endodontic therapy relies on chemotactic biological cues to recruit the body’s own stem/progenitor cells to sites of pulp injury. These cues guide the migration of stem/progenitor cells from periapical tissues to the damaged area, enabling the regeneration of dental pulp. Biomaterial scaffolds enriched with growth factors provide structural support and biochemical signals, creating a conducive microenvironment for cell adhesion, proliferation, and differentiation. Together, these elements work synergistically to restore the vitality and function of the dental pulp–dentin complex.

Cell homing relies on the recruitment of endogenous stem/progenitor cells to sites of injury, orchestrated by a complex interplay of signaling molecules, ECM components, and the cellular microenvironment [12,13]. This process is a hallmark of regenerative mechanism in various tissues and is pivotal in achieving functional restoration in pulp regeneration. The fundamental mechanisms of cell homing include the chemotaxis of stem/progenitor cells from periapical tissues and the application of biomaterial scaffolds enriched with signaling molecules (Figure 1). These mechanisms facilitate not only the effective recruitment and retention of stem/progenitor cells but also promote their differentiation and integration into functional tissue.

### 3.1. Chemotaxis

Central to the cell homing process is chemotaxis, a biological phenomenon where cells migrate along a gradient of chemotactic signals [12,13]. Chemokines, growth factors, and cytokines create these gradients by being released into the periapical tissues. The following signaling molecules are known to play a significant role in chemotaxis (Table 1).

#### 3.1.1. Stromal Cell-Derived Factor-1

Stromal cell-derived factor-1 (SDF-1), also known as chemokine (C-X-C motif) ligand 12 (CXCL12), is a chemoattractant that interacts with its receptor CXCR4 to support cell mobilization and homing [19,20,21]. It plays a crucial role in the migration and proliferation of various cell types, including hematopoietic stem cells and mesenchymal stem cells (MSCs) [22,23,24]. SDF-1 promotes the activity of CD31-/CD146- side population (SP) cells, aiding in the regeneration of dental pulp-like tissue with capillaries and nerves in dogs following transplantation into root canals [25,26]. In addition, studies have demonstrated that localized upregulation of SDF-1 significantly enhances cell homing efficiency [12,21,24,27]. Furthermore, inhibiting the SDF-1/CXCR4 interaction leads to reduced migration of stem cells to target tissues [21].

#### 3.1.2. Platelet-Derived Growth Factor

Platelet-derived growth factor (PDGF), released by platelets, is highly effective in promoting cell proliferation and angiogenesis as well as chemotaxis [28,29,30,31]. It exists in four homodimer isoforms (PDGF-AA, PDGF-BB, PDGF-CC, and PDGF-DD) and one heterodimer (PDGF-AB) [32,33,34]. These PDGF dimers interact with two cell surface receptors, PDGFRα and PDGFRβ, which form dimers prior to binding specific PDGF isoforms [33,34]. PDGFRα/α dimers bind to PDGF-AA, -BB, and -CC, while PDGFRα/β dimers bind to PDGF-AB, -BB, -CC, and -DD [32,33,34]. In addition, PDGFRβ/β dimers interact with PDGF-BB and -DD [32,33,34]. The biological effects of PDGF are determined by the expression levels and configurations of PDGFR dimers on target cells. PDGF induces the chemotaxis and proliferation of MSCs at injury sites, where platelets release PDGF to attract immune cells and support early wound healing. While PDGFs stimulate cell proliferation and dentin matrix protein synthesis, they inhibit alkaline phosphatase activity and mineralized tissue formation, showing varying effects on odontoblast differentiation based on the PDGF isoform [35,36,37]. It has been shown that PDGF-bb with a collagen matrix supports pulp revascularization and healing of periapical lesions in an endodontically treated molar [38].

#### 3.1.3. Fibroblast Growth Factor

Fibroblast growth factor (FGF), notably FGF2, is a critical factor in cell migration, proliferation, and differentiation during embryonic development, wound healing, and regeneration of the pulp–dentin complex [39,40,41,42]. There are 23 identified members of the FGF family in humans, each with distinct functions [43], and FGF2 is significant in the context of dental tissue repair and regeneration [43]. FGF2 exerts its biological effects by interacting with heparan sulfate (HS) and heparan sulfate proteoglycans present on cell surfaces [44,45]. This interaction is critical for signal transduction through the four known FGF receptors (FGFR1 to FGFR4) [43]. It has been demonstrated that FGF2 promotes the migration of dental pulp cells into three-dimensional collagen gels in transwell migration assays [46]. This aspect illustrates the ability of FGF2 to recruit cells to sites of injury, thereby facilitating the repair and regeneration process. In addition, FGF2 stimulates the proliferation of dental pulp cells without triggering differentiation at early stages, which is critical for initial repair efforts. When combined with transforming growth factor-beta 1 (TGFβ1), FGF2 further enhances the differentiation of these cells into odontoblast-like cells, thereby supporting dentin regeneration and the development of dentin structures [47]. Nagy et al., in their randomized controlled trial, found that FGF2 combined with the blood clot scaffold induced periapical healing in immature necrotic maxillary central incisors, although no significant difference was observed compared to the group treated with a blood clot alone [48].

#### 3.1.4. Transforming Growth Factor-β

Transforming growth factor-β (TGFβ) plays a significant role in regulating cell migration [49,50,51,52,53,54,55]. The chemotactic effect of TGF β involves multiple mechanisms, including the ERK/MAPK signaling pathway [53], the downregulation of Rho GTPase activating proteins (ARHGAPs) [54], and cytoskeletal reorganization [55]. The effects of TGFβ are highly variable and depend significantly on the cell type and tissue context in which it operates. TGFβ1 has also been shown to enhance cell proliferation and increase ECM production in dental pulp tissue cultures [56]. Moreover, TGFβ1 promotes odontoblastic differentiation in dental pulp cells, indicating its crucial role in dentinogenesis [47]. TGFβ1 plays a pivotal role in the immune response during dental pulp injuries, modulating inflammatory processes that are critical for tissue recovery and homeostasis [57,58,59]. This multifaceted involvement of TGFβ1 highlights its importance in both regenerative and pathological contexts within dental tissues. TGFβ exists in three isoforms (TGFβ1, TGFβ2, and TGFβ3) and remains inactive in a large latent complex until proteolytic cleavage [60,61]. Once activated, it binds to the Type II receptor (TGFβRII), recruits the Type I receptor (TGFβRI), and triggers phosphorylation of SMAD2 and SMAD3 [62,63]. The resulting activated SMAD complex translocates to the nucleus, where it governs downstream gene transcription crucial for diverse cellular functions [64].

#### 3.1.5. Summary: Signaling Molecules in Chemotaxis

Several signaling molecules play pivotal roles in the chemotactic processes that govern stem cell migration and differentiation. For instance, SDF-1 significantly facilitates the mobilization and migration of MSCs by binding to its specific receptor, CXCR4 [19,20,21]. Similarly, PDGF is instrumental in promoting angiogenesis while also aiding in the recruitment of MSCs, which is vital for the regeneration of the pulp–dentin complex [28,29,30,31]. Furthermore, FGF enhances both cell migration and differentiation, which are essential for effective pulp–dentin repair and regeneration [39,40,41,42]. TGFβ also plays a crucial role, as it regulates cell migration and stimulates odontoblastic differentiation, thereby contributing to tissue regeneration [47,50,51,52,53,54,55].

### 3.2. Stem/Progenitor Cells Mobilized from Periapical Tissues

Cell homing approaches hinge on the recruitment of stem and progenitor cells from periapical tissues. Notable sources within the periapical area include mainly stem cells from the apical papilla (SCAPs) [64,65,66,67], inflammatory periapical progenitor cells (iPAPCs) [68], bone marrow mesenchymal stem cells (BMMSCs) [69,70], and periodontal ligament stem cells (PDLSCs) [71,72]. Each type has distinct characteristics and clinical implications, contributing significantly to advancements in regenerative endodontics and overall dental health (Table 2).

#### 3.2.1. Stem Cells from the Apical Papilla

SCAPs are mesenchymal stem cells located in the immature apical region of developing teeth, where they play a crucial role in root development and pulp regeneration [64]. They share a common origin with dental pulp stem cells (DPSCs) but exhibit distinct biological characteristics that set them apart [73]. SCAPs were first identified in 2006 when the cells were isolated from the apical papilla of human third molars and recognized their MSC properties, which include clonogenicity and differentiation into odontoblast-like and osteoblast-like cells [74,75,76]. Interestingly, SCAPs are highly resistant to chronic inflammation and may survive apical periodontitis due to the low density of vasculature in the apical papilla and the nutrient supply from the highly vascularized surrounding dental follicle [77].

SCAPs are characterized by their expression of specific surface markers typical of MSCs, such as STRO-1, CD146, and the unique SCAP-specific marker CD24 [67,78,79]. Compared to DPSCs, SCAPs exhibit higher proliferation rates and enhanced tooth tissue regeneration abilities, potentially linked to elevated levels of survivin and telomerase expression in SCAPs [67]. However, key odontogenic markers such as dentin sialophosphoprotein, matrix extracellular phosphoglycoprotein, TGFβ RII, FGFR3, VEGFR-1, and FGFR1 show lower expression levels in SCAPs compared to DPSCs [80,81]. This suggests distinct functional roles for SCAPs and DPSCs during dental tissue formation and repair. The in vivo differentiation capability of SCAPs was demonstrated based on the regeneration of the pulp and dentin tissues in immunodeficient mice when combined with appropriate scaffolds [81]. SCAPs have been shown to differentiate into odontoblasts, osteoblasts, adipocytes, and neurons [67,74,76,79,82,83,84]. Moreover, the secretion of brain-derived neurotrophic factor (BDNF) from SCAPs has been recognized as critical for neuronal growth in vitro, further establishing their potential for neural regeneration [85]. Semi-quantitative RT-PCR analyses confirmed that gene expression levels of BDNF, glial cell-derived neurotrophic factor, and angiopoietin-1 in SCAPs are higher than those seen in other dental MSCs [86].

#### 3.2.2. Inflamed Periapical Progenitor Cells

iPAPCs are a type of mesenchymal stem/progenitor cell found within inflamed periapical tissues, which commonly occur in response to pulp necrosis or periapical infection [68]. Importantly, while iPAPCs originate from inflamed tissues, they retain the ability to respond positively to regenerative stimuli, indicating a capacity for functional tissue regeneration. One of the main characteristics of iPAPCs is their multilineage differentiation potential, which allows them to develop into multiple cell types relevant to restorative processes in dental tissues [68]. Furthermore, the inflammatory microenvironment may influence their behavior, enhancing their ability to migrate and localize to sites of injury. The harnessing of iPAPCs from periapical tissues presents an important strategy for cell homing-based regenerative endodontics.

#### 3.2.3. Bone Marrow Mesenchymal Stem Cells

BMMSCs are a specialized subset of mesenchymal stem cells located in the bone marrow microenvironment [69,70]. They are characterized by their surface markers, including CD73, CD90, and CD105, which are crucial for their identification and isolation [87]. BMMSCs exhibit a unique capacity for self-renewal, allowing them to proliferate extensively while maintaining their stem cell characteristics over multiple cell divisions [88,89]. Another feature of BMMSCs is their heterogeneity, suggesting that different subpopulations within BMMSC cultures may exhibit varying differentiation potentials or responses to specific stimuli [90,91]. This heterogeneity is instrumental in their ability to adapt to different environments and fulfill various functional roles in tissue repair and regeneration. Under appropriate conditions, BMMSCs can differentiate into several lineages, such as osteogenic, chondrogenic, and adipogenic lineages [89,92]. BMMSCs can also differentiate into odontoblasts [93,94,95], and this ability is particularly important when aiming to restore dental pulp and dentin integrity. Interestingly, BMMSCs also exhibit some ability to differentiate into neural cell types [96], which may contribute to restoring nerve function in the dental pulp, facilitating better integration of dental tissues following regeneration. BMMSCs can differentiate into osteoblasts, essential for supporting bone formation and responding to bone loss typically observed in periapical diseases [89,92]. This differentiation is critical in promoting the healing of periapical lesions and the establishment of a healthy periodontal environment. The mobilization of BMMSCs to the root canal space presents a promising therapeutic potential for a cell homing strategy.

#### 3.2.4. Periodontal Ligament Stem Cells

PDLSCs are a type of mesenchymal stem cell located in the periodontal ligament surrounding the roots of teeth [71,72]. They are characterized by the expression of specific surface markers, including CD90, CD146, and STRO-1, which are indicative of their stem-like properties [97,98]. These cells differentiate into various cell types, including osteoblasts, chondrocytes, and fibroblasts [99,100]. Moreover, PDLSCs possess immunomodulatory functions that can help mitigate inflammatory responses, making them particularly valuable in the context of regenerative therapies where inflammation is a common impediment to healing [101,102,103]. PDLSCs have the capability to differentiate into odontoblast-like and osteoblast-like cells, contributing to dentin regeneration and periapical bone healing [104]. Recruiting periodontal ligament stem cells from periapical tissues represents a promising avenue in regenerative endodontics.

#### 3.2.5. Summary: Stem/Progenitor Cell Sources

The success of regenerative therapies is heavily reliant on the sources of stem/progenitor cells. SCAPs are particularly beneficial due to their high proliferation rates and inflammation resistance, making them pivotal in dentin–pulp complex regeneration [73,74,75,76,77]. iPAPCs found within inflamed periapical tissues exhibit multilineage differentiation capabilities, contributing significantly to restorative processes [68]. BMMSCs are known for their adaptability to diverse environments, aiding in both bone and pulp regeneration [89,92,93,94,95]. Furthermore, PDLSCs possess immunomodulatory functions alongside their potential for odontoblastic differentiation [101,102,103,104].

### 3.3. Biomaterial Scaffolds Containing Signaling Molecules

The use of biomaterial scaffolds incorporating signaling molecules can facilitate the regeneration of the pulp–dentin complex. There are various types of biomaterial scaffolds that contain biological molecules, including blood clots, platelet-rich plasma (PRP), platelet-rich fibrin (PRF), dehydrated human amnion–chorion membrane (dHACM), exosome-embedded scaffolds, functionalized biomaterials, and ECM-based scaffolds utilized in pulp regeneration (Table 3).

#### 3.3.1. Blood Clot

Blood clots have become increasingly recognized for their potential as natural scaffolds in regenerative endodontics, particularly in the management of immature teeth diagnosed with pulp necrosis [105,106]. Utilizing the body’s intrinsic healing mechanisms, blood clots offer a unique solution for facilitating pulp regeneration. The key to utilizing blood clots as scaffolds lies in their composition. Blood clots are rich in fibrin, a protein that provides a three-dimensional network capable of supporting cell adhesion and migration [107]. This fibrous structure allows for the hosting of various cell types, including MSCs and progenitor cells, which play critical roles in the regenerative process [107,108]. In addition, by acting as a reservoir for signaling molecules and growth factors, blood clots promote cell proliferation, angiogenesis, and overall healing of the tissue [109]. The application of blood clots in regenerative endodontics has illustrated promising outcomes in various clinical studies [110]. The protocol typically involves creating a blood clot within the root canal after disinfecting the canal and inducing bleeding. This generated clot acts as a scaffold where stem/progenitor cells can proliferate and differentiate into a variety of cells, such as odontoblasts, endothelial cells, fibroblasts, and nerve cells, which may contribute to pulp tissue regeneration.

Clinical studies have shown comparable success rates for blood clots relative to other scaffold materials, such as PRP and PRF, in achieving goals such as root development, resolution of periapical lesions, and restoration of vitality in necrotic teeth [111]. In essence, the use of blood clots acts not only to restore tooth structure but also to stabilize the healing process throughout various stages of tissue regeneration, leveraging the body’s natural biomaterials and processes for optimal results. Moreover, blood clots have the advantage of being derived from the patient’s own body, minimizing risks of immune rejection and adverse reactions associated with synthetic materials [112]. This autologous nature enhances their appeal as a scaffold choice while facilitating faster integration with surrounding tissue.

Despite the potential of blood clots as scaffolds, several challenges persist. Variability in clot formation efficiency can occur due to factors such as limitations in the volume of evoked bleeding and the individual biological response to injury. Effective induction of bleeding remains critical, as inadequate bleeding can lead to insufficient clot formation and ultimately compromise the regenerative process. In addition, while blood clots do provide several growth factors, the overall variation in the concentrations and types of growth factors released can lead to differences in regenerative outcomes across different patients. The variability in histological outcomes has been observed, often resulting in the formation of periodontal tissue rather than fully functional pulp tissue.

#### 3.3.2. Platelet-Rich Plasma

PRP has become an important scaffold due to its ability to deliver high concentrations of growth factors that support regenerative processes [113]. PRP is prepared by centrifuging a patient’s blood to concentrate platelets, followed by activation to promote degranulation and the release of growth factors [114]. The biological efficacy of PRP is primarily attributed to its rich composition of growth factors and cytokines that play a vital role in various aspects of tissue healing and regeneration, such as PDGF, vascular endothelial growth factor (VEGF), and TGFβ [115]. These growth factors released from activated platelets create a favorable microenvironment that not only influences the migration and proliferation of stem cells but also drives their differentiation into various pulp cells, necessary for regenerating the pulp–dentin structure [116].

Clinical studies of PRP as a scaffold in regenerative endodontics report similar outcomes to those achieved with blood clots, often with improved apical closure and dentin wall thickening [110,117]. The growth factors present in PRP may enhance cellular activities, such as the proliferation and differentiation of stem cells, leading to faster and more effective apical closure than might be achieved through blood clot scaffolds alone. Studies have reported that the application of PRP can lead to thickening of the dentin walls while promoting root development, thereby reducing the risk of future complications associated with weaker root structures [110,117]. This thickening process is essential for the longevity of revitalized teeth. Despite its considerable advantages, the application of PRP in clinical settings presents several challenges. The efficacy of PRP can vary significantly depending on individual patient factors (such as age and health) [118,119], collection techniques, and the specifics of the centrifugation process. Such variability can lead to differences in growth factor concentrations and overall effectiveness. In addition to potential patient discomfort during procurement of PRP, it involves specific equipment and techniques that may not be widely available in all dental practices, potentially limiting its accessibility to practitioners and patients alike.

#### 3.3.3. Platelet-Rich Fibrin

PRF, developed as a second-generation platelet concentrate, has gained attention as a scaffold offering a simpler preparation protocol than PRP, making it a desirable option for clinicians [120,121]. PRF consists of a dense fibrin matrix enriched with platelets and leukocytes, which not only retains growth factors but also promotes a controlled release over time, enhancing the regenerative potential [121,122]. The controlled release nature of these growth factors from the fibrin matrix ensures sustained stimulation of the healing processes, which is advantageous for conditions requiring prolonged regenerative activity [121,122,123].

Clinical studies have indicated that PRF can effectively stimulate healing and pulp regeneration, showing comparable outcomes to PRP and blood clots [110,117]. However, like other platelet concentrates, the benefits of PRF must be balanced against considerations of preparation complexity and cost.

#### 3.3.4. Dehydrated Human Amnion–Chorion Membrane

The dehydrated amnion–chorion membrane (dHACM) has emerged as a promising scaffold due to its excellent biocompatibility and anti-inflammatory properties [124]. This membrane is derived from human placental tissue and provides a natural extracellular matrix conducive to cell attachment, proliferation, and differentiation [124,125]. It naturally provides ECM that facilitates cell attachment, proliferation, and differentiation [124]. In regenerative endodontic treatment, dHACM may improve healing by offering a supportive framework for stem cells and by delivering a wide array of growth factors, including TGFβ, FGF, PDGF, VEGF, placental growth factor, hepatocyte growth factor, and granulocyte colony-stimulating factor [126,127,128,129]. dHACM also contains diverse cytokines, such as interleukins and tissue inhibitors of metalloproteinases, which help regulate the immune response to minimize inflammation [128]. By controlling the inflammatory process, these cytokines prevent excessive tissue damage and encourage more efficient healing—an essential advantage in regenerative applications, where curbing chronic inflammation can lead to superior outcomes. Adding to its regenerative promise, dHACM carries several key ECM proteins, including collagen, laminin, fibronectin, and glycosaminoglycans [126,127,128]. These components provide structural reinforcement, serving as both a scaffold for cell attachment and migration and as a conducive environment for crucial cellular events, such as proliferation and differentiation. In addition to bolstering the membrane’s physical properties, these ECM proteins contribute to biochemical signaling, a fundamental process in tissue development and repair. Notably, Kim and Solomon have shown that dHACM promoted dental pulp regeneration by inducing greater odontoblast cell lining on native dentin and enhancing periapical healing in a canine model [130]. Despite these encouraging results, challenges remain, such as variability in the source and processing of the membrane, which can affect its properties and clinical effectiveness.

#### 3.3.5. Exosome-Embedded Biomaterials

Exosomes, small extracellular vesicles that play a crucial role in intercellular communication, have garnered considerable interest as innovative scaffolds due to their ability to carry signaling molecules and growth factors essential for tissue regeneration [131]. They enhance cell proliferation and differentiation by mediating paracrine signaling in the local microenvironment of the dental pulp [131,132,133].

Exosomes are nanoscale extracellular vesicles, typically ranging from 30 to 150 nanometers in diameter, which are released from nearly all cell types into the extracellular environment [134]. They are formed from the internal endosomal membrane system and play a critical role in intercellular communication by transporting various bioactive molecules, including proteins, lipids, and nucleic acids, to neighboring or distant cells [135,136]. The composition and function of exosomes are influenced by their cellular origin, suggesting that exosomes derived from different types of cells can carry specialized cargo that can affect target cells in distinct ways [134,137]. In the context of regenerative endodontics, exosomes are primarily derived from MSCs. These exosomes encapsulate growth factors, cytokines, and microRNAs that can modulate various biological processes, including inflammation, cell survival, and tissue repair [131,132,133,134]. Exosomes have been shown to enhance the migration and proliferation of dental-derived stem cells [131,132,138,139]. For instance, DPSCs can secrete exosomes that promote the recruitment of MSCs to the site of injury, effectively augmenting the regenerative process [140]. The mobilization of MSCs from surrounding tissues increases the pool of regenerative cells and accelerates healing. Exosomes can also modulate the inflammatory response, leading to an improved healing environment [139,141]. Exosomes derived from DPSCs can release bioactive molecules that promote anti-inflammatory cytokine production, thereby ameliorating excessive inflammation and encouraging tissue repair [139,141]. In addition, exosomes can stimulate angiogenesis, the formation of new blood vessels, which is essential for sustaining newly formed tissues [139,142]. Studies have demonstrated that exosomes carry proangiogenic factors that enhance endothelial cell proliferation and tube formation [139,142,143]. Moreover, exosomes can influence the differentiation of stem cells into odontoblasts [139,144]. By delivering signaling molecules, exosomes can activate various intracellular pathways that dictate the cellular fate of stem cells within the dental pulp.

Exosomes derived from human DPSCs (hDPSCs) have been successfully used in experimental studies to promote the regeneration of the pulp–dentin complex in animal models. Chen et al. have demonstrated that a hydrogel containing small extracellular vesicles (exosomes) derived from hDPSCs, whether cultured with or without lipopolysaccharide, enhanced structural features of regenerated pulp tissue in pulpectomized root canals of Sprague-Dawley rats [145]. In this study, the highest level of vascularization was observed when BMSCs were transplanted with exosomes from lipopolysaccharide-preconditioned hDPSCs [145]. Zhuang et al., using a subcutaneous root fragment implantation model, have shown that transplanting rat BMMSC and human SCAP-derived exosomes embedded in a gelatin sponge into emptied root canals resulted in the formation of dentin–pulp-like tissue [146]. The tissue included newly organized odontoblasts along the regenerated dentin. Further research is required to elucidate the efficacy of exosomes in cell homing-based regenerative endodontic therapy. It is also important to determine the best methods for exosome isolation, characterization, and application in regenerative therapies to maximize their therapeutic potential.

#### 3.3.6. Biomaterials Functionalized with Growth Factors

The functionalization of biomaterial scaffolds with growth factors is a critical strategy to enhance their regenerative potential [147]. Growth factors are naturally occurring proteins that regulate various cellular activities, including growth, proliferation, differentiation, and tissue healing. By incorporating these bioactive molecules into scaffolds, the regeneration of dentin and pulp tissues can be significantly improved. Various types of biomaterials have been studied, with categories including natural polymers, such as collagen and chitosan, and synthetic polymers, such as poly(lactic acid) (PLA), polyglycolic acid (PGA), poly(lactic-co-glycolic acid) (PLGA), and polycaprolactone (PCL) for dental pulp regeneration [8,105,148]. Recently, hybrid scaffolds have been developed and evaluated for pulp regeneration. Li et al. demonstrated that the gelatin–fibrin scaffolds enhanced cell migration, odontogenic differentiation, and dentin formation with fibrin, improving their structural and functional properties [149]. Loukelis et al. developed kappa-carrageenan, chitosan, and gelatin scaffolds for pulp regeneration [150]. The scaffolds exhibited high alkaline phosphatase activity and odontogenic marker expression, especially when potassium chloride was added [150]. Bordini et al. created chitosan–calcium aluminate scaffolds and assessed their cell homing potential in an in vitro microchip platform [151]. The scaffolds demonstrated strong chemotactic and bioactive properties, including cell migration, odontoblastic differentiation, and mineralized matrix deposition [151]. These biomaterials can be functionalized with growth factors such as bone morphogenetic proteins (BMPs), VEGF, FGF, PDGF, nerve growth factor (NGF), and granulocyte colony-stimulating factor (G-CSF). A recent study by Noohi et al. developed a hydrogel scaffold functionalized with platelet-rich fibrin extract to support the regeneration of the pulp–dentin complex [152]. This in vitro study utilized methacrylated chitosan and collagen to create a light-curable bicomponent hydrogel. This scaffold demonstrated enhanced chemotaxis, biomineralization, and vascularization, which are critical for cell homing-based pulp regeneration [152]. In a study by Han et al., collagen hydrogels loaded with DPSCs and growth factors were injected into the pulp chamber of tooth slices [153]. When implanted subcutaneously in immunodeficient mice, the combination of low-stiffness collagen with VEGF and high-stiffness collagen with BMP2 enhanced pulp-like tissue formation, vascularization, and dentin regeneration [153].

Several animal studies have explored the use of biomaterial scaffolds that are functionalized with growth factors for the regeneration of the pulp–dentin complex. A minipig study by He et al. demonstrated that a collagen gel with BMP7 and/or Wnt3a into the pulpectomized root canal space of mandibular incisors regenerated neurovascular connective tissues and dentin-like tissue [154]. The scaffold delivering Wnt3a alone or in combination with BMP7 resulted in the formation of tubular dentin with odontoblasts within the regenerated dentin [154]. In an animal study by Kim et al., dental pulp-like tissue was regenerated when collagen scaffolds functionalized with growth factors were placed in endodontically treated root canals of extracted human teeth and implanted into the dorsum of mice [155]. The growth factors used in this study were FGF2, VEGF, or PDGF, combined with a basal set of NGF and BMP7. Iohara et al. conducted a study using a collagen scaffold with SDF-1, total pulp cells with SDF-1, or SDF-1 with CD105+ cells to regenerate pulp tissue in canine incisors [156]. All experimental groups successfully regenerated vascularized pulp-like tissue. However, the transplantation of pulp CD105+ cells with SDF-1 resulted in significantly higher levels of pulp tissue regeneration compared to the other groups [156]. In another study by Iohara et al., the collagen scaffolds impregnated with G-CSF were transplanted into pulpectomized teeth in dogs with or without DPSCs. This approach successfully regenerated pulp tissue, including vascularization and innervation [157]. Notably, the combination of DPSCs and G-CSF produced a significantly greater amount of regenerated pulp–dentin complexes compared to the use of G-CSF alone or DPSCs alone.

#### 3.3.7. Extracellular Matrix-Based Scaffolds

The ECM serves as a critical component of tissues, playing a pivotal role in providing structural support while influencing cellular behavior through biochemical and biomechanical cues [158,159]. In regenerative medicine, ECM-derived scaffolds, including decellularized matrices from sources like dental pulp, have emerged as viable solutions for mimicking tissue-specific microenvironments [160,161,162,163]. These scaffolds are particularly promising for regenerating the pulp–dentin complex, as they preserve essential signaling molecules and proteins. Studies have shown that decellularized dental pulp matrices enable the differentiation of stem cells into odontogenic phenotypes [164,165]. Innovative methods, such as the decellularization of human dental pulp from third molars and swine pulp ECM, have shown great efficacy [166,167]. For instance, scaffolds from decellularized swine dental pulp successfully supported the proliferation and differentiation of DPSCs, resulting in the formation of odontoblast-like cells and mineralized tissue [167]. In addition, hydrogels derived from decellularized ECM have been explored, enhancing cell viability and migration [168]. A recent study by Elnawam et al. demonstrated that hydrogels derived from decellularized bovine pulp ECM, with or without hyaluronic acid, retained bioactive properties, with hyaluronic acid-enriched ECM hydrogel showing sustained growth factor release of TGF-β1, VEGF, and FGF2 [169]. However, challenges remain regarding the stability of certain scaffolds, with approaches like cross-linking being suggested to address issues of degradation in culture environments.

#### 3.3.8. Summary: Biomaterial Scaffolds for Pulp Regeneration

Biomaterial scaffolds play a fundamental role in providing both structural and biochemical support to facilitate regeneration. Various types of scaffolds are utilized, including blood clots, PRP, PRF, dHACM, exosome-embedded biomaterials, biomaterials functionalized with growth factors, and extracellular matrix-based scaffolds. Blood clots are a cost-effective and autologous option but exhibit variability in their effectiveness [107,108,109,110]. PRP enhances the delivery of growth factors [113,114,115,116], while PRF offers sustained release, thus optimizing the healing environment [121,122,123]. dHACM represents another biomaterial scaffold that is anti-inflammatory and conducive to cell attachment and differentiation [124,125,126,127,128,129]. In addition, exosomes are innovative carriers of signaling molecules that can accelerate tissue regeneration and improve healing environments [131,132,133,134]. Lastly, functionalizing biomaterials with essential growth factors significantly enhances their regenerative capabilities [147,148,149,150,151,152,153,154,155,156,157], while extracellular matrix-based scaffolds derived from dental pulp can effectively mimic tissue-specific environments for better cellular integration and growth [158,159,160,161,162,163,164,165,166,167,168,169].

## 4. Cell Homing-Based Regenerative Endodontics

Regenerative endodontics is an evolving field that offers the potential to restore the structural and functional integrity of damaged dental tissues, particularly in cases involving pulp necrosis or apical periodontitis. Among the numerous approaches being explored, cell homing stands out as a promising therapeutic intervention that harnesses the body’s inherent regenerative capacity [6,7]. This technique emphasizes the recruitment of endogenous cells to the root canal space to foster tissue regeneration, a process that is influenced by a variety of experimental conditions, biomaterials, and signaling molecules. Animal studies using cell homing-based regenerative endodontics are summarized in Table 4.

### 4.1. Animal Models

A variety of animal models have been utilized in the exploration of cell homing-based regenerative endodontics, with dogs being the predominant choice due to their anatomical and physiological resemblance to human dental structures [130,170,171,172,173,174,175,176,177,178,180,181,182,183,184,185,187,189,190,191,193,195,197,198,200,201,202]. Other models, such as ferrets [179,192,194], rats [188], pigs [154,196,199], and sheep [186], have also contributed to a comprehensive understanding of the principles of this regenerative technique. In these studies, various tooth types, including premolars, incisors, and molars, were examined, often focusing on cases that presented with preexisting infections. This emphasis on infection reflects the need to simulate clinical conditions that practitioners encounter in human patients, thereby enhancing the relevance of the findings.

### 4.2. Biomaterial Scaffolds

The selection and application of biomaterial scaffolds are critical to the success of any regenerative procedure. Various scaffolds, including blood clots [130,170,171,172,173,174,175,176,177,178,179,180,181,182,183,184,185,186,187,188,189,190,191,192,193,194,195,196,197,200,201,202], collagen [130,154,170,171,172,173,175,185,186,190,192,202], PRP [176,179,181,182,197], PRF [187,195,200], and injectable hydrogels [154,196,198,199,202] have been employed in different experimental protocols. These scaffolds create a supportive framework for cell recruitment and tissue formation. Notably, comparative studies have illustrated that while some scaffolds, such as blood clots and PRP, show similar efficacy in terms of facilitating apical closure and tissue regeneration [176,182,197], the intricacies of scaffold compatibility can influence overall outcomes.

### 4.3. Exogenous Signaling Molecules

The incorporation of signaling molecules like basic fibroblast growth factor (bFGF) [174], stromal cell-derived factor-1α (SDF-1α) [180], and bone morphogenetic proteins (BMPs) [154] into scaffolds has been a focal point for enhancing regenerative outcomes. These molecules play pivotal roles in promoting the recruitment and differentiation of endogenous stem cells toward pulp and dentin tissue. For instance, BMP7 has been associated with enhanced bone-like tissue formation, while SDF-1α has shown significant promise in fostering pulp-like tissue regeneration while minimizing the formation of mineralized tissue [154,180]. Wnt3a, or the combination of Wnt3a and BMP7, has been demonstrated to promote the formation of tubular dentin and pulp-like tissue, including the development of nerve structures [154].

### 4.4. The Presence of Previous Infection

Studies consistently observed the formation of cementum-like, bone-like, and periodontal ligament-like tissues in both infected [130,170,171,172,173,174,175,176,177,178,179,180,181,182,183,184,185,186,187,189,191,193,195,197,200,201,202] and non-infected groups [154,177,188,190,192,194,196,198,199]. However, the histological findings suggest that infection does not significantly impede the ability of regenerative strategies to achieve these outcomes. The presence of previous infection did not significantly affect the formation of vital connective tissues in most studies. In general, studies showed that previous infection did not significantly alter the overall success of regenerative procedures. Both infected and non-infected models achieved comparable results in apical closure, mineralized tissue deposition, and root maturation [177].

### 4.5. Histological Outcomes

The histological evaluations of the regenerated state post-therapy reflect varied yet interesting patterns. Regenerated tissues often included cementum-like, bone-like, and periodontal ligament-like tissues. Mineralized tissue deposition and apical closure were common histological outcomes. Despite varied experimental conditions, significant differences in outcomes were rare between different scaffolds or signaling molecules. Furthermore, the observed root maturation and formation of dentin-like tissues substantiate the restorative capabilities of the cell homing approach. However, some scaffolds—particularly those incorporating hyaluronic acid or fibrin—have been linked to elevated inflammatory responses [196,202], highlighting a crucial element in scaffold design.

The comparison between blood clots and PRP has revealed interesting insights into their roles as scaffolds for regenerative endodontics. Most studies suggest that there is no significant difference in the outcomes related to apical closure or mineralized tissue deposition when utilizing blood clot scaffolds versus PRP [176,182,197]. This finding indicates that both options can effectively facilitate basic regenerative processes. Injectable scaffolds present another noteworthy development in the field [154,196,198,199,202]. These scaffolds have demonstrated effectiveness comparable to traditional blood clots while providing more controlled delivery of growth factors [154,199]. The ability to inject these materials into complex root canal morphology allows for better adaptation and filling of the space. Consequently, injectable scaffolds can optimize the regenerative process by ensuring that growth factors are released in a controlled manner, which may enhance the overall regenerative potential of the treatment.

## 5. Challenges and Future Directions

### 5.1. Challenges of Cell Homing Techniques

#### 5.1.1. Limited Recruitment Efficiency

One of the foremost limitations of cell homing in regenerative endodontics is the limited recruitment efficiency of stem cells. Often, the recruitment of these stem cells is suboptimal, requiring high concentrations of chemotactic factors to facilitate their migration into the pulp–dentin complex. This inefficiency not only complicates the procedures but can also lead to inconsistent results across different patients and clinical scenarios.

#### 5.1.2. Microenvironment Optimization

Another challenge is the limited control over the microenvironment within the root canal space, where factors such as infection and tissue inflammation can significantly impair the regenerative process [203]. Effective disinfection before applying cell homing strategies remains critical. However, traditional disinfecting agents can be cytotoxic to stem cells if not carefully managed [204]. The balance between effective disinfection and maintaining a supportive environment for stem cell viability is delicate and often shifts according to the protocols applied, emphasizing the need for standardized clinical guidelines.

#### 5.1.3. Patient Variability

Clinical translation of cell homing techniques also faces significant challenges due to the variability in patient biology and the intrinsic complexity of dental pulp tissues. There exists a wide range of physiological differences among patients that can influence their response to treatment protocols. Factors such as age and individual immune responses can lead to inconsistent outcomes, making the standardization of treatment protocols a difficult task. The scientific community has yet to establish universal guidelines that account for these variations, complicating the widespread adoption of these regenerative strategies in clinical practice.

#### 5.1.4. Variability of Outcomes in Clinical Scenarios

A significant challenge in the implementation of cell homing techniques is the variability of outcomes observed across different clinical scenarios [48,204,205,206,207,208,209,210,211]. This inconsistency arises from several interconnected factors, including biological heterogeneity among patients, the condition of the pulp and periapical microenvironment, and variability in procedural techniques. Differences in intrinsic factors, such as the levels of growth factors and chemokines, which play a critical role in mediating the cell homing process, can significantly diminish the efficacy of these therapies in various individuals. In addition, the presence of preexisting infections and inflammation can severely impede stem cell migration and integration. Even when effective disinfection techniques are employed, residual bacteria may persist, leading to complications during the regenerative process. Furthermore, the interactions between stem cells and their surrounding infected or inflamed microenvironment may predispose treatments to failure, thereby contributing to inconsistent results. Variations in how practitioners execute cell homing strategies can also introduce disparities in effectiveness. The specific methods used for root canal disinfection, as well as the choice of disinfecting agents, can lead to differences in stem cell viability and recruitment efficiency. Moreover, the lack of consensus regarding optimal protocols for cell homing applications, including scaffold selection and growth factor delivery, creates variability in clinical approaches. This inconsistency can result in differing outcomes even under similar theoretical conditions, ultimately complicating the adoption of these innovative regenerative strategies in clinical practice.

### 5.2. Future Directions

To address these limitations effectively, future research and development must adopt a multifaceted approach. The advancement of biomaterials used in cell homing strategies is crucial. Ongoing research into bioactive scaffolds that can encapsulate and release growth factors in a controlled, sustained manner is likely to enhance the regenerative environment, thereby improving the recruitment efficiency of stem cells. Furthermore, establishing standardized protocols based on patient factors such as the degree of periapical inflammation, age, and immune responses is indispensable for ensuring reproducibility and reliability in regenerative therapies. Comprehensive guidelines detailing the appropriate use of biomaterials, chemotactic factor concentrations, and disinfection methods will foster consistency in clinical results. By addressing the challenges linked to patient variability and developing clear treatment frameworks, practitioners can work towards more predictable outcomes in regenerative endodontics.

## 6. Conclusions

The field of regenerative endodontics has made remarkable strides, particularly through the adoption of cell homing-based approaches. These innovative techniques capitalize on the body’s intrinsic regenerative capabilities, serving as a promising alternative to conventional treatments, such as root canal therapy. By deploying signaling molecules, harnessing endogenous stem/progenitor cells, and utilizing biomaterial scaffolds, cell homing strategies aim to restore both the structural and functional integrity of the dental pulp–dentin complex, leading to biologically superior and more sustainable outcomes.

The integration of chemotactic factors—including SDF-1, PDGF, FGF, and TGFβ—has proven effective in enhancing the recruitment, migration, and differentiation of stem/progenitor cells such as SCAPs, BMMSCs, iPAPCs, and PDLSCs. Mobilized from periapical tissues, these cells are instrumental in regenerating vascularized, innervated, and mineralized pulp-like tissue. Furthermore, biomaterials, including blood clots, PRP, PRF, dHACM, exosome-embedded scaffolds, biomaterials functionalized with growth factors, and ECM-based scaffolds have demonstrated their potential to foster cellular activities and enhance the microenvironment required for effective tissue regeneration.

Nonetheless, despite the promising results from animal models and preclinical studies, several challenges remain. Issues such as limited recruitment efficiency, the necessity for optimization of the regenerative microenvironment, and patient-specific variability create hurdles for successful clinical application. The variability in treatment outcomes highlights the pressing need for standardized protocols that are tailored to individual patient conditions and therapeutic objectives.

Future research should prioritize the refinement of biomaterial scaffolds, the enhancement of controlled release mechanisms for growth factors, and strategies to address the variability inherent in patient responses. Moreover, the establishment of universal clinical guidelines and rigorous translational studies is vital for ensuring the reliability and scalability of these regenerative practices within daily dental care. Cell homing-based regenerative endodontic therapy possesses transformative potential for preserving natural tooth vitality while reducing dependence on devitalizing procedures. With ongoing innovation and clinical validation, this shift in paradigm can redefine standards of care in dental pulp therapy, significantly improving patient outcomes and advancing the field of regenerative dentistry.

## Figures and Tables

**Table 1:** The role and mechanisms of key signaling molecules in chemotaxis for cell homing-based regenerative endodontics.

Signaling Molecules	Key Role	Mechanisms
Stromal Cell-Derived Factor-1 (SDF-1) [19,20,21,22,23,24,25,26,27]	Supports cell mobilization and homing by interacting with CXCR4. Promotes migration and proliferation of hematopoietic and mesenchymal stem cells. Enhances regeneration of pulp-like tissue.	Interacts with CXCR4 to establish chemotactic gradients. Upregulated SDF-1 enhances the efficiency of cell homing.
Platelet-Derived Growth Factor (PDGF) [28,29,30,31,32,33,34,35,36,37,38]	Promotes cell proliferation, angiogenesis, and chemotaxis. Supports pulp revascularization and healing of periapical lesions.	PDGF dimers bind to PDGFR receptors, stimulating MSC chemotaxis and early wound healing.
Fibroblast Growth Factor (FGF) [39,40,41,42,43,44,45,46,47,48]	Facilitates cell migration, proliferation, and differentiation. Critical for repair and regeneration of the pulp–dentin complex.	FGF2 interacts with heparan sulfate proteoglycans for signal transduction, aiding in cell recruitment and differentiation.
Transforming Growth Factor-β (TGFβ) [49,50,51,52,53,54,55,56,57,58,59,60,61,62,63,64]	Regulates cell migration, proliferation, and ECM production. Enhances odontoblastic differentiation and modulates the immune response.	Activates SMAD signaling pathways to regulate gene transcription. Plays a multifaceted role in tissue recovery and regeneration.

**Table 2:** Stem/progenitor cells in cell homing-based regenerative endodontics.

Stem/Progenitor Cells	Characteristics	Clinical Implications
Stem Cells from the Apical Papilla (SCAPs) [64,73,74,75,76,77,78,79,80,81,82,83,84,85,86]	Located in the immature apical region of developing teeth. High proliferation rates and multipotent differentiation potential. Highly resistant to chronic inflammation.	Regenerating dentin–pulp-like complex; potential for neural tissue regeneration.
Inflamed Periapical Progenitor Cells (iPAPCs) [68]	Found in inflamed periapical tissues. Multilineage differentiation potential.	Potential for restorative processes in dental tissues; responds positively to regenerative stimuli.
Bone Marrow Mesenchymal Stem Cells (BMMSCs) [69,70,87,88,89,90,91,92,93,94,95,96]	Specialized subset located in the bone marrow. Self-renewal and high heterogeneity, adapting to various environments.	Restoring dental pulp and dentin integrity; contributes to healing periapical lesions and bone formation.
Periodontal Ligament Stem Cells (PDLSCs) [97,98,99,100,101,102,103,104]	Located in the periodontal ligament surrounding the roots of teeth. Immunomodulatory functions and multilineage differentiation potential.	Potential for pulp regeneration; forms new dentin and enhances the structural integrity of the tooth.

**Table 3:** Biomaterial scaffolds for cell homing-based regenerative endodontics.

Biomaterial Scaffolds	Key Characteristics	Biological Effects
Blood Clot [105,106,107,108,109,110,111,112]	Natural scaffold rich in fibrin. Support for cell adhesion, migration, and differentiation. Reservoir for signaling molecules.	Facilitates pulp regeneration in immature teeth with necrosis; promotes angiogenesis and healing.
Platelet-Rich Plasma [113,114,115,116,117,118,119]	Concentrated platelets with high levels of growth factors. Enhancement of cell proliferation and differentiation.	Improves apical closure, dentin wall thickening, and root development in regenerative endodontics.
Platelet-Rich Fibrin [120,121,122,123]	Dense fibrin matrix with sustained release of growth factors. Simpler preparation compared to PRP.	Stimulates healing and pulp regeneration and is effective for conditions requiring prolonged regenerative activity.
Dehydrated Human Amnion–Chorion Membrane [124,125,126,127,128,129,130]	Derived from human placenta; ECM proteins and cytokines. Excellent biocompatibility and anti-inflammatory properties.	Enhances healing and cell attachment and supports proliferation and differentiation in regenerative endodontics.
Exosome-Embedded Biomaterials [131,132,133,134,135,136,137,138,139,140,141,142,143,144,145,146]	Bioactive molecules, growth factors, and microRNAs. Modulation of inflammation and enhancement of cell proliferation.	Promotes pulp–dentin complex regeneration, accelerates healing, and enhances angiogenesis.
Biomaterials Functionalized with Growth Factors [147,148,149,150,151,152,153,154,155,156,157]	Growth factors such as BMP, VEGF, and FGF are incorporated into scaffolds for tissue regeneration.	Regenerates dentin-like and pulp-like tissues and supports vascularization and cell differentiation.
Extracellular Matrix-Based Scaffolds [158,159,160,161,162,163,164,165,166,167,168,169]	Decellularized tissues mimic natural ECM for cell attachment and differentiation.	Provides structural support and biochemical cues and facilitates the regeneration of functional pulp-like tissues.

**Table 4:** Summary of animal studies using cell homing-based regenerative endodontics.

Study	Animal Models	Tooth Types	Presence of Previous Infection	Biomaterial Scaffolds	Exogenous Signaling Molecules	Time after Cell Homing Procedure	Main Histological Findings
Thibodeau et al., 2007 [170]	Dog	60 premolars from 6 dogs	Yes	Blood clot or collagen or blood clot + collagen	N/A	3 months	No statistical difference in histological outcomes was observed among the experimental groups. Vital tissue was present in 29.3% of the roots, hard tissue deposition on root dentin was noted in 43.9% of the roots, and apical closure occurred in 54.9% of the roots.
Wang et al., 2010 [171]	Dog	60 premolars from 6 dogs	Yes	Blood clot or collagen or blood clot + collagen	N/A	3 months	Further histological analysis was conducted based on Thibodeau et al. (2007). Cementum-like tissue (intracanal cementum), bone-like tissue (intracanal bone), and periodontal ligament-like tissue were observed in the root canal space.
Yamauchi et al., 2011 [172]	Dog	60 premolars from 6 dogs	Yes	Blood clot or cross-linked collagen scaffold (CCS) or blood clot + CCS	N/A	3.5 months	Dentin-associated mineralized tissue (DAMT) and bony islands (Bis) were identified in the root canal. Significantly more mineralized tissues were observed in the groups with CCS.
Yamauchi et al., 2011 [173]	Dog	60 premolars from 6 dogs	Yes	Blood clot or cross-linked collagen scaffold (CCS) or blood clot + CCS	N/A	3.5 months	Further histological analysis was conducted based on Yamauchi et al. (2011). DAMT resembles cementum and differs from dentin and bone. BIs have characteristics similar to bone tissue.
Tawfik et al., 2013 [174]	Dog	108 premolars from 9 dogs	Yes	Blood clot or blood clot + injectable scaffold coated with bFGF	bFGF	1 week 3 weeks 3 months	The in-growth of connective tissue and cementoid tissue were observed. The injectable scaffold with growth factor was found to be no more effective than a blood clot in promoting tooth development.
Yoo et al. 2014 [175]	Dog	30 premolars from 3 dogs	Yes	Blood clot + collagen scaffold sponge (CSS) or blood clot + CSS soaked with conditioned medium (CM) from preameloblasts	N/A	3 months	CSS and CM treatment resulted in significantly enhanced root maturation and the development of more pulp-like and osteodentin-like tissues. Some CM-treated samples showed newly formed dentin-like tissue.
Zhang et al., 2014 [176]	Dog	27 premolars from 3 dogs	Yes	Blood clot or platelet-rich plasma (PRP)	N/A	3 months	No significant differences were found between blood clots and PRP regarding apical closure, pulp-like tissue formation, and new hard tissue formation on the canal wall.
Khademi et al., 2014 [177]	Dog	36 teeth (maxillary incisors and mandibular premolars) from 3 dogs	Yes (20 teeth) No (10 teeth)	Blood clot	N/A	3 months 6 months	The cementum-like and bone-like tissues, along with highly vascular granulation tissue, were observed. No significant difference was found between the necrotic-infected group and the vital group.
Saoud et al., 2015 [178]	Dog	17 teeth (maxillary and mandibular anteriors) from 2 dogs	Yes	Blood clot	N/A	3 months	Ingrowth of loose connective tissue or fibrous tissue continuous with the periodontal ligament was noted. Cementum-like and bone-like tissues, as well as ingrowth of alveolar bone, were also observed.
Torabinejad et al., 2015 [179]	Ferret	24 canines from 6 ferrets	Yes	Blood clot + gelfoam or PRP	N/A	3 months	Bone-like, cementum-like, and connective tissues were observed. There was no significant difference in hard tissue deposition and apical narrowing between the blood clot + gelfoam group and the PRP group.
Yang et al., 2015 [180]	Dog	8 premolars from 2 dogs	Yes	Blood clot or blood clot + SDF-1α-loaded silk fibroin scaffold	SDF-1α	3 months	Intracanal connective tissue and mineralized tissue were observed. In the SDF-1α group, more connective tissue and less mineralized tissue, similar to normal pulp, was noted, compared to the blood clot group.
Stambolsky et al., 2016 [181]	Dog	40 premolars from 4 dogs	Yes	Blood clot or PRP	N/A	6 months	The highest level of vital tissue and hard tissue deposition, similar to cementum and bone, was observed in teeth disinfected with sodium hypochlorite and tri-antibiotic paste, followed by the application of PRP.
Moradi et al., 2016 [182]	Dog	28 premolars from 2 dogs	Yes	Blood clot or PRP	N/A	1 month 3 months	Soft connective tissue, blood vessels, and hard mineralized tissue were observed. No significant difference was found between the blood clot and PRP in the formation of vital tissue.
Pagliarin et al., 2016 [183]	Dog	40 premolars from 4 dogs	Yes	Blood clot	N/A	7 months	The cementum-like, bone-like, and periodontal ligament-like tissues were found. The teeth treated with propolis paste exhibited a higher amount of vital tissue compared to those treated with triple antibiotic paste.
Dianat et al., 2016 [184]	Dog	20 teeth (single rooted and double rooted) from 3 dogs	Yes	Blood clot or plasma rich in growth factors (PRGFs)	N/A	6 months	The granulation tissue, along with cellular cementum and bone-like tissue, was observed. No significant difference was noted between the blood clot and PRGF.
El Ashry et al., 2016 [185]	Dog	144 premolars from 12 dogs	Yes	Blood clot or blood clot + collagen scaffold	N/A	2 weeks 6 weeks 3 months	There is no significant difference between blood clots and blood clots with collagen scaffolds in connective tissue ingrowth, new hard tissue formation, and apical closure.
Altaii et al., 2016 [186]	Sheep	8 incisors from 4 sheep	Yes	Blood clot + absorbable collagen dressing	N/A	6 months	Fibrovascular connective tissue, cementum-like tissue deposition, and root maturation were observed.
Zhou et al., 2017 [187]	Dog	24 premolars from 3 dogs	Yes	Blood clot or blood clot + platelet-rich fibrin (PRF)	N/A	3 months	Cementum-like tissue, periodontal ligament-like tissue, and bone-like tissue were observed. No significant difference was found between the blood clot and the blood clot with PRF in root development.
Moreira et al., 2017 [188]	Rat	29 molars from 29 rats	No	Blood clot	N/A	4 weeks	The immature connective tissue, along with blood vessels, nerves, and odontoblast-like cells, was formed under the stimulation of photobiomodulation therapy.
Palma et al., 2017 [189]	Dog	96 premolars from 4 dogs	Yes	Blood clot or blood clot + sodium hyaluronate/chitosan or blood clot + pectin/chitosan	N/A	13 weeks	The bone-like tissue, cementum, and periodontal ligament were observed. Blood clots resulted in the highest amount of mineralized tissue in the root canal compared to the two chitosan scaffolds.
Alqahtani et al., 2018 [190]	Dog	8 teeth (molars and premolars) from 2 dogs	No	Blood clot or collagen sponges or decellularized swine dental pulp extracellular matrix (DP-ECM)	N/A	8 weeks	All three groups exhibited cellular infiltrations and intracanal mineralization. The DP-ECM group specifically showed the development of dentin sialoprotein-expressing dental pulp-like tissue.
Bottino et al., 2019 [191]	Dog	10 premolars from 1 dog	Yes	Blood clot	N/A	3 months	Osteodentin with cellular inclusions was observed, along with the apical root closure.
Bucchi et al., 2019 [192]	Ferret	24 canines from 6 ferrets	No	Blood clot + collagen sponge with or without preameloblast-conditioned medium	N/A	2 months	Vascularized connective tissue, intracanal Sharpey’s fibers, and cementum-like tissue were observed, regardless of the use of the conditioned medium.
He et al., 2019 [154]	Pig	58 mandibular incisors from 10 minipigs	No	Collagen gel or collagen gel + Wnt3a or collagen gel + BMP7 or collagen gel + Wnt3a + BMP7	Wnt3a BMP7	3 months	In the BMP7 group, excessive bone-like tissue was observed. The Wnt3a and Wnt3a + BMP7 groups exhibited newly formed tubular dentin, as well as pulp-like tissue and nerves.
Abbas et al., 2020 [193]	Dog	120 premolars from 8 dogs	Yes	Blood clot or blood clot + chitosan loaded with demineralized bone matrix or blood clot + chitosan loaded with dexamethasone	N/A	1 week 1 month 3 months	The formation of periodontal ligament-like tissue and apical hard tissue was observed. No significant difference was noted between blood clots and chitosan scaffolds in hard tissue deposition.
Alexander et al., 2020 [194]	Ferret	32 canines from 8 ferrets	No	Blood clot or SynOss putty scaffold	N/A	3 months	The blood clot group exhibited a root canal space filled with bone-like tissue. In contrast, only one tooth in the SynOss putty group showed any newly formed tissue.
El Halaby et al., 2020 [195]	Dog	126 premolars from 9 dogs	Yes	Blood clot or PRF	N/A	1 month 2 months 3 months	The PRF group exhibited significantly higher vital tissue and hard tissue formation compared to the blood clot group.
Jang et al., 2020 [196]	Pig	24 premolars from 4 minipigs	No	Blood clot or blood clot + gelatin-based matrix (GM) or blood clot + fibrin-based matrix (FM)	N/A	3 months	Pulp-like tissue and mineralized tissue deposition with root maturation were observed in all experimental groups. However, greater inflammatory infiltrates were found in the FM group.
Xi et al., 2020 [197]	Dog	54 teeth from 6 dogs	Yes	Whole blood or PRP	N/A	3 months	There was no difference between the two scaffold groups in apical closure, mineralized tissue deposition, and connective tissue ingrowth.
Kim and Solomon 2021 [130]	Dog	16 premolars from 2 dogs	Yes	Blood clot or blood clot + collagen membrane (CM) or blood clot + amnion–chorion membrane (ACM)	N/A	3 months	In the ACM group, a greater amount of intracanal fibrous connective tissues and odontoblast-like cell linings were observed compared to the blood clot and blood clot with CM groups. Intracanal mineralized tissue was only found in the blood clot and CM groups.
Siddiqui et al., 2021 [198]	Dog	12 incisors from 2 dogs	No	Nano-porous peptide scaffolds (hydrogel) (SLan, Sled)	N/A	28 days	Angiogenic SLan hydrogels promoted the formation of pulp-like tissue, nerves, and dentin-like tissue with odontoblast-like cells. In contrast, dentinogenic Sled hydrogels led to disorganized tissue structure.
Wang et al., 2021 [199]	Pig	Teeth from 5 minipigs	No	Injectable alkaline hydrogel with gelatin microspheres (MS-gel) or collagen gel	N/A	3 months	Vascularized pulp-like tissue, dentin-like tissue, and nerves were found in the pH 10 MS-gel group, while ectopic mineralized tissue was observed in the collagen gel group.
Elnawam et al., 2024 [200]	Dog	16 premolars from 2 dogs	Yes	Blood clot or injectable PRF or hyaluronic acid (HA) or bovine dental pulp-derived extracellular matrix (P-ECM)	N/A	3 months	New tissue ingrowth was found in all experimental groups. The P-ECM group exhibited well-organized connective tissue. The blood clot and PRF groups showed a higher amount of mineralized tissue compared to the HA and P-ECM groups.
Fouad et al., 2024 [201]	Dog	72 root canals (premolars) from 3 dogs	Yes	Blood clot	N/A	1 month 2 months 3 months	The newly formed hard tissue, resembling tubular dentin lined with odontoblasts, was observed. The photobiomodulation group induced greater vital tissue infiltration and hard tissue deposition.
Abdelsalam et al., 2024 [202]	Dog	16 premolars from 2 dogs	Yes	Blood clot or blood clot + hyaluronic acid hydrogel (HA) or blood clot + collagen	N/A	3 months	Pulp-like tissue with newly formed dentin was observed. No significant difference was found among the experimental groups in tissue ingrowth. However, more inflammatory cells were noted in the HA group.

## Data Availability

Not applicable.
